# Utilization patterns of insulin therapy and healthcare services among Japanese insulin initiators during their first year: a descriptive analysis of administrative hospital data

**DOI:** 10.1186/s12913-016-1264-2

**Published:** 2016-01-12

**Authors:** Shunya Ikeda, Bruce Crawford, Masayo Sato

**Affiliations:** 1 International University of Health and Welfare, Ohtawara, Japan; 2 IMS Japan KK, Tokyo, Japan; 3 Adelphi Values LLC, Tokyo, Japan; 4 Eli Lilly Japan KK, Kobe, Japan

**Keywords:** Diabetes, Insulin, Utilization pattern, Healthcare resource utilization, Japan

## Abstract

**Background:**

Type 2 diabetes poses an increasing healthcare burden in Japan. Although insulin treatment has diversified in recent years, the literature on the utilization of healthcare services among patients with type 2 diabetes undergoing different insulin therapy regimens is scarce. The current study aimed to characterize the real-world insulin treatment patterns and associated utilization of healthcare services among patients with type 2 diabetes who initiated insulin therapy during the study period.

**Methods:**

We examined data from a hospital-based database consisting of administrative and laboratory data from 121 acute-phase hospitals throughout Japan from April 2008 to August 2012. Patients diagnosed with type 2 diabetes and receiving continuous insulin therapy, defined by three insulin claims or more, were included in the analysis.

**Results:**

Of the 2,145 insulin initiators, at initiation 46.5 % received rapid-acting insulin alone, 36.6 % received an intensive regimen, 11.4 % received long-acting insulin alone, and 5.5 % received pre-mixed insulin alone. Patients treated with rapid-acting insulin alone were older, experienced more comorbid conditions, had lower HbA1c, and more often had initiated their insulin treatment at inpatient admission, compared to patients treated with other types of insulin. Inpatient admission was more common and longer for patients taking rapid-acting insulin and an intensive regimen than those taking long-acting or pre-mixed insulin, and most were readmitted within 1 year. Utilization of outpatient clinics was approximately once per month, and emergency department visits were observed to be rare.

**Conclusions:**

This retrospective observational descriptive study found varied treatment and healthcare service utilization patterns, as well as disparities in patient characteristics across insulin regimens. Future research should assess the basis for these various utilization patterns associated with insulin to conduct robust analyses of clinical and economic outcomes.

**Electronic supplementary material:**

The online version of this article (doi:10.1186/s12913-016-1264-2) contains supplementary material, which is available to authorized users.

## Background

Diabetes poses a significant and increasing healthcare burden. The Japanese Ministry of Health, Labour and Welfare conducted the National Health and Nutrition Survey in 2012 and found that nearly one quarter of individuals were either diabetic or prediabetic (11.4 % for diabetes and 12.7 % for prediabetes) [1]. It is estimated that 9.5 million individuals in Japan had type 2 diabetes mellitus (T2DM) in 2012 [1], a gradual increase from 8.9 million in 2007 [2]. Recently, more individuals sought treatment for their diabetes, with 2.7 million^1^ diagnosed patients visiting a clinic or hospital for diabetes in 2011 compared to an estimated 2.1 million in 1999 [3].

The increasing prevalence of diabetes and the growing number of patients receiving treatment represents a significant economic burden. The total healthcare expenditure for diabetes was 1,209 billion JPY—4.3 % of the total healthcare expenditure in 2012 [4]. Just as prevalence rates have increased steadily, the overall healthcare expenditure on diabetes has been rising, with an increase of nearly 100 billion JPY from 2005 to 2012, from 1,117 billion JPY to 1,209 billion JPY [4, 5].

Alongside the increases in prevalence and cost, insulin treatment has diversified in recent years. With respect to initiation and adjustment of insulin, the Japan Diabetes Society (JDS) treatment guideline [6] recommends diet and exercise therapies as the first step in treating T2DM, followed by one type of oral hypoglycemic agent or insulin in small doses and then combination therapy, should the preceding steps be unsuccessful [6]. Various factors including patients’ age, level of obesity, the state of liver and kidney function, insulin secretory capacity, and the degree of insulin resistance should be determined prior to starting pharmacological treatment [6]. Previous studies reveal the use of different types and combinations of insulin for diabetes treatment [7], as well as a diverse role of insulin in different settings for a variety of patients with diabetes mellitus [8]. Today, patients can initiate their insulin therapy in more personalized, patient-tailored manners, ranging from long-acting insulin only to supplementing with oral hypoglycemic agents to a combination of rapid- and long-acting insulin for an intensive regimen [9].

Despite a large number of studies on T2DM, little information exists on the real-world treatment patterns and on the associated healthcare service use among Japanese insulin initiators. By examining an administrative hospital database containing electronic health records from acute-phase hospitals throughout Japan, the current study aimed to characterize the real-world utilization patterns of healthcare services for patients with T2DM on different types of insulin therapy regimens over the 1-year period following initiation of insulin therapy.

## Methods

### Data sources and data collection

An automated hospital-based database obtained from hospital electronic information systems, developed by Medical Data Vision Co., Ltd (MDV), was utilized. The database contained administrative and laboratory data from 121 acute-phase hospitals throughout Japan, among which 25 had less than 200 beds, 75 had between 200 and 499 beds, and 21 had 500 beds or more. This anonymous database had similar age and gender distribution to that of national patient statistics [3] and included information on drug prescriptions, medical procedures, surgeries, laboratory results such as HbA1c, diagnosis codes, age, gender, date of medical service, department, and inpatient/outpatient status [10]. Although HbA1c was reported with the JDS system in the database because it was widely used in Japan at the time of data collection, all HbA1c values in the current study were converted to the National Glycohemoglobin Standardization Program (NGSP) system with the formula recommended by JDS (NGSP = JDS + 0.4 [11]). Data collected between April 1, 2008, and August 31, 2012, were analyzed. The closest value to their index date was used as their baseline HbA1c value.

### Population

Patients were included in the study based on the following criteria: 1) diagnosed with T2DM, identified with the International Classification of Disease 10th revision (ICD-10) codes of E11 (non-insulin dependent diabetes mellitus), E12 (malnutrition-related diabetes mellitus), or E14 (unspecified diabetes mellitus), with no codes for E10 (type 1 diabetes mellitus); 2) received insulin, identified using ICD-10 code Z79.4 and drug codes (Additional file 1: Table S1); and 3) were at least 18 years old when they first received insulin. It was also required that patients had at least 18 months of data because insulin-naïve patients were identified by having no record of insulin claim during the 6-month period prior to their first insulin claim (=index date) and the 1-year post index period was used for the analysis. In addition, patients who did not have three or more insulin claims during the 1-year post index period, as well as patients who were inpatients on their index date without one or more insulin claims after discharge, were excluded. To ensure we had true insulin initiators, patients with a small number of insulin claims were excluded because they may have already been receiving most of their care at a hospital not included in the database and it was possible that they only visited a hospital in this database for acute treatment and received insulin during their inpatient stay. Because the database did not track patients outside of the hospitals included, strict criteria were used to limit the number of misclassifications, which also limited the effective sample size for analyses.

Insulin treatments were classified into the following four groups based on the types and intensity of therapy prescribed over the 1-year post index period: long-acting insulin, rapid-acting insulin, pre-mixed insulin, and intensive regimen groups. The long-acting group included patients who received long acting insulin (e.g., insulin glargine, insulin detemir) or intermediate-acting insulin (e.g., isophane) and did not have a claim for any other types of insulin for 100 days after their long-acting insulin claim. The 100-day gap was used because patients can be provided up to 90 days of insulin at one time. The rapid-acting insulin group included patients who received rapid-acting insulin (e.g., lispro, aspart, gulisine) or regular insulin (e.g., insulin neutral [regular]) and did not have a claim for any other types of insulin for 100 days after their rapid-acting insulin claim. The pre-mixed insulin group included patients who received a prepared combination of rapid- and long-acting insulin and did not have a claim for any other types of insulin for 100 days after their pre-mixed insulin claim. The intensive regimen group included patients who received a combination of rapid-acting, long-acting, and/or pre-mixed insulin. If patients had claims for multiple types of insulin during the 1-year post index period and if the number of days between the two adjacent claims was less than 100 days, an assumption was made that these patients belonged to the intensive regimen group. On the other hand, if the adjacent claims were for different types of insulin and if they were prescribed with a gap of 100 days or more between them, an assumption was made that these patients first initiated with one type of insulin and later switched to another type of insulin. Patients with gaps of more than 100 days were considered to be intermittent users if they used insulin again at a later date or were considered dropped out if no further claims for insulin were found.

### Statistical analysis

Descriptive analyses were performed on the study population for patient characteristics, treatment patterns, and healthcare service utilization. Continuous variables were described by presenting the mean, standard deviation, median, minimum, and maximum. Categorical variables were described by presenting the number and percentage of patients in each category. If HbA1c was not measured at index, the closest HbA1c value prior to the index date was used for the blood glucose analyses. Data reduction and descriptive statistics were performed using SAS software version 9.1.

### Ethics statement

The study was approved by the Ethics Committee of the International University of Health and Welfare (#12-190).

## Results

### Selected patients

**Fig. 1:**
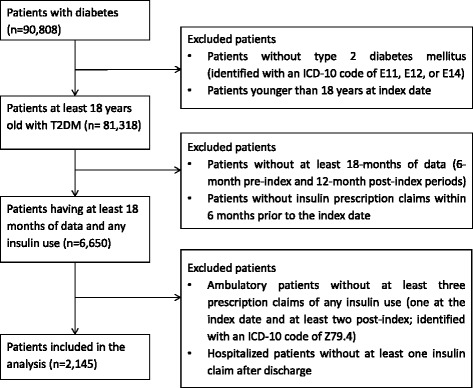
Patient selection chart

Sample selection and attrition are detailed in Fig. 1. Of 90,808 patients with diabetes mellitus visiting the hospitals in the database between April 1, 2008, and August 31, 2012, a total of 81,318 were at least 18 years old and had a diagnosis of T2DM. Of 6,650 patients who had data for both the 6-month pre-index period required to determine previous treatment history and the 1-year post-index period required for the analysis, 2,145 patients were determined to be insulin initiators who received most of care at the hospitals in the database and included in the analysis. To reduce misclassification of patients, patients who did not have three or more insulin claims during the 1-year post index period were excluded.

### Insulin regimen at initiation

**Fig. 2:**
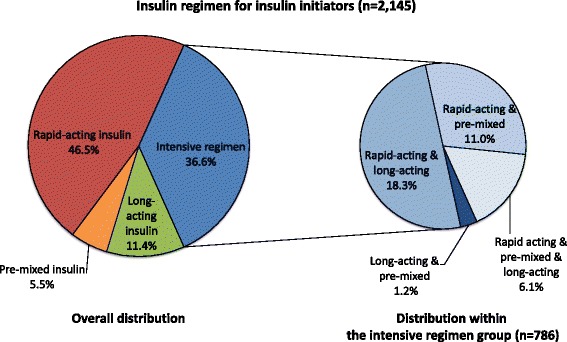
Distribution of insulin regimen type at initiation

Of the 2,145 patients who initiated and continued insulin therapy, more than half (63.4 %; *N =* 1,359) were received a simple regimen with one type of insulin (rapid-acting, pre-mixed, or long-acting insulin only), whereas the remainder (36.6 %; *N =* 786) received a combination of multiple insulin types (an intensive regimen). Approximately half of patients were in the rapid-acting insulin group (46.5 %; *N =* 997), and fewer patients were in the long-acting insulin group (11.4 %; *N =* 244) or the pre-mixed insulin group (5.5 %; *N =* 118). Among those receiving an intensive regimen, four types of insulin combinations were identified (Fig. 2). Almost all patients (96.7 %; *N =* 760) in the intensive regimen group used rapid-acting insulin as a part of a combination therapy with long-acting and/or pre-mixed insulin.

### Patient characteristics

The population characteristics for patients who initiated insulin are summarized by insulin group in Table 1. Patients in the rapid-acting insulin group were the oldest, with a mean age of 72.0 years (SD, 11.2 years), compared with those receiving other types of insulin, with means ranging from 65.2 to 66.7 years. More than half of the patients were male in all insulin groups. Patients receiving rapid-acting insulin had the lowest HbA1c (7.4 %)^2^ [11], and those receiving long-acting insulin had the highest HbA1c (9.4 %)^3^ [11].

**Table 1:** Population demographic and clinical characteristics (*N =* 2,145)

Baseline Patient Characteristic (*N =* 2,145)	Long-acting	Pre-mixed	Rapid-acting	Intensive
	(*N =* 244)	(*N =* 118)	(*N =* 997)	(*N =* 786)
Age (years)				
Mean (±SD)	65.2 (±12.3)	66.3 (±12.9)	72.0 (±11.2)	66.7 (±12.3)
Range	23-95	24-98	22-105	19-95
Gender, n (%)				
Male	158 (64.8)	78 (66.1)	609 (61.1)	477 (60.7)
HbA1c (%)^a^				
Mean	9.4	8.0	7.4	9.1
Median	9.2	7.8	7.0	9.1
Treatment initiation setting, n (%)				
Inpatient	40 (16.4)	47 (39.8)	907 (91.0)	573 (72.9)
Outpatient	204 (83.6)	71 (60.2)	90 (9.0)	213 (27.1)
Pre-index antidiabetic medication (OAD), n (%)				
Yes	225 (92.2)	84 (71.2)	752 (75.4)	623 (79.3)
DPP-4 inhibitors	127 (52.0)	38 (32.2)	419 (42.0)	316 (40.2)
Alpha glucosidase inhibitors	143 (58.6)	47 (39.8)	374 (37.5)	309 (39.3)
Biguanide	128 (52.5)	30 (25.4)	232 (23.3)	261 (33.2)
Gurinido	40 (16.4)	9 (7.6)	147 (14.7)	128 (16.3)
SU	187 (76.6)	47 (39.8)	507 (50.9)	427 (54.3)
TZD	106 (43.4)	22 (18.6)	179 (18.0)	169 (21.5)
Single-pill combination	10 (4.1)	-	8 (0.8)	5 (0.6)

^a^HbA1c was available only for those with HbAc1 measurement data

^a^HbA1c was reported with the Japan Diabetes Society (JDS) system in the database; all HbA1c values in this study were converted to the National Glycohemoglobin Standardization Program (NGSP) system with the formula recommended by JDS (NGSP = JDS + 0.4 [11])

SD, standard deviation; DPP-4, dipeptidyl peptidase-4 inhibitors; SU, sulfonylureas; TZD, thiazolidinediones

 Nearly all patients (92.2 %; *N =* 225) in the long-acting insulin group and the majority of patients in each group (71.2 % [*N =* 84], 75.4 % [*N =* 752], and 79.3 % [*N =* 623] for the pre-mixed insulin, rapid-acting insulin, or intensive regimen group, respectively) received at least one oral antidiabetic drug (OAD) or exenatide (injectable) prior to insulin initiation. The most commonly used OAD across all insulin initiators was sulfonylureas (SU), followed by dipeptidyl peptidase-4 (DPP-4) inhibitors and alpha glucosidase inhibitors.

Comorbid conditions are summarized in Table 2. Overall, fewer comorbid conditions were observed in the long-acting insulin group compared with the other groups. Less than half of all insulin initiators were diagnosed with hypertension (40.6 %; *N =* 870) and approximately one quarter were diagnosed with nephropathy (26.6 %; *N =* 571).

**Table 2:** Patient comorbid conditions (*N =* 2,145)

Comorbid Condition (*N =* 2,145)	Overall (*N =* 2,145)	Long-acting (*N =* 244)	Pre-mixed (*N =* 118)	Rapid-acting (*N =* 997)	Intensive (*N =* 786)
Comorbid conditions, n (%)					
Hypertension	870 (40.6)	40 (16.4)	42 (35.6)	475 (47.6)	313 (39.8)
Nephropathy	571 (26.6)	31 (12.7)	39 (33.1)	301 (30.2)	200 (25.4)
Ischemic heart disease	398 (18.6)	28 (11.5)	18 (15.3)	212 (21.3)	140 (17.8)
Heart failure	331 (15.4)	15 (6.1)	12 (10.2)	200 (20.1)	104 (13.2)
Cerebrovascular disease	300 (14.0)	9 (3.7)	11 (9.3)	172 (17.3)	108 (13.7)
Neuropathy	189 (8.8)	13 (5.3)	7 (5.9)	100 (10.0)	69 (8.8)
Arthropathy	171 (8.0)	8 (3.3)	-	109 (10.9)	54 (6.9)
Depression	59 (2.8)	2 (0.8)	-	36 (3.6)	21 (2.7)
Peripheral vascular disease	54 (2.5)	5 (2.0)	1 (0.8)	26 (2.6)	22 (2.8)
Skin complications	37 (1.7)	1 (0.4)	2 (1.7)	15 (1.5)	19 (2.4)
Retinopathy	34 (1.6)	1 (0.4)	1 (0.8)	15 (1.5)	17 (2.2)
Myocardial infarction	28 (1.3)	1 (0.4)	2 (1.7)	12 (1.2)	13 (1.7)
Hypoglycemia	25 (1.2)	-	-	18 (1.8)	7 (0.9)
Hyperglycemia	12 (0.6)	1 (0.4)	-	7 (0.7)	4 (0.5)
Ketoacidosis	7 (0.3)	-		-	7 (0.9)
Oral complications	1 (0.0)	-	-	1 (0.1)	-

### Insulin utilization patterns

As seen in Fig. 2, most intensive regimens combined rapid-acting insulin and/or other insulin types; the majority of insulin initiators (81.9 %; *N =* 1,757) used rapid-acting insulin. Nearly all patients receiving rapid-acting insulin (91.0 %; *N =* 907) and the majority of patients receiving an intensive regimen (72.9 %; *N =* 573) initiated insulin in an inpatient setting. Conversely, those patients receiving long-acting and pre-mixed insulin (83.6 % and 60.2 %, respectively) initiated at an outpatient setting (Table 1).

Table 3 describes insulin utilization patterns among insulin initiators during their first year of insulin treatment. More than half of patients receiving long-acting insulin (63.5 %; *N =* 155) and pre-mixed insulin (55.1 %; *N =* 65), as well as nearly half of patients receiving an intensive regimen (42.9 %; *N =* 337), were using insulin consistently for at least 1 year after insulin initiation. Almost all patients receiving rapid-acting insulin (94.4 %; *N =* 941) used insulin intermittently or stopped using insulin within 1 year (defined as at least 100 days between two insulin claims or between the last one and the end of the observation period). When used intermittently, insulin was used for a few months, on average, with a shorter median duration of insulin use for those receiving pre-mixed insulin (72 days) and rapid-acting insulin (71 days) and a longer median duration of insulin use for those receiving long-acting insulin (154 days) and an intensive regimen (107 days).

**Table 3:** Insulin utilization patterns by insulin type (*N =* 2,145)

Insulin Use (*N =* 2,145)	Long-acting (*N =* 244)	Pre-mixed (*N =* 118)	Rapid-acting (*N =* 997)	Intensive (*N =* 786)
Used consistently^a^, n (%)	155 (63.5)	65 (55.1)	56 (5.6)	337 (42.9)
Used intermittingly or dropped out^b^, n (%)	89 (36.5)	53 (44.9)	941 (94.4)	449 (57.1)
Duration of insulin use, days				
Mean (±SD)	150.7 (±108.7)	121.8 (±112.8)	108.1 (±104.5)	132.1 (±102.1)
Median	154.0	72.0	71.0	107.0

^a^ Patients did not have more than 100 days between two adjacent insulin treatments or more than 100 days between the last insulin treatment and the end of the 1-year post-index period

^b^ Patients had more than 100 days between two adjacent insulin treatments or more than 100 days between the last insulin treatment and the end of the 1-year post-index period

SD, standard deviation

Table 4 describes the insulin treatment modification patterns over the 1-year post-index period based on claims in their first month. Less than half of patients who were prescribed one type of insulin on their first insulin claim modified their insulin regimen by switching or adding a different type of insulin at least once over the 1-year post-index period (37.7 %, 44.7 %, and 34.0 % for long-acting, pre-mixed, and rapid-acting insulin, respectively). The median number of days for modification of the initial treatment after the date they received their first insulin claim was 86, 28, and 17 days for long-acting, pre-mixed, and rapid-acting insulin, respectively. On the other hand, the majority (84.4 %; *N =* 184) of patients who had multiple types of insulin on their first insulin claim modified their insulin regimen after a median of 15 days. One patient had a record for unspecified type of insulin on the first insulin claim; therefore, this patient was excluded from the treatment modification analysis only.

**Table 4:** Insulin treatment modification by initial insulin type from first claim (*N =* 2,145)

	Insulin type prescribed on the first claim				
	Long-acting insulin only	Pre-mixed insulin only	Rapid-acting insulin only	Intensive insulin	Unspecified insulin
N (%)	363 (16.9)	190 (8.9)	1373 (64.0)	218 (10.2)	1 (0.0)
Switched or added an insulin type at least once over the 1-year period, n (%)	137 (37.7)	85 (44.7)	467 (34.0)	184 (84.4)	NA
Days between index and first insulin switch or addition^a^					
Mean (±SD)	119.0 (±118.7)	91.1 (±113.2)	71.5 (±100.7)	54.3 (±82.1)	NA
Median	86	28	17	15	NA

^a^Only patients with at least one insulin type switch over the 1-year index period were included

SD, standard deviation; NA, Not applicable

### Healthcare service utilization

Data on healthcare service utilization are summarized in Table 5. Nearly all patients receiving rapid-acting insulin (95.5 %; *N =* 952) and the majority of patients receiving an intensive regimen (87.9 %; *N =* 691) were hospitalized at some point during the first year of insulin treatment, whereas only half of (55.1 %; *N =* 65) and one third of patients (32.8 %; *N =* 80) receiving pre-mixed and long-acting insulin, respectively, were hospitalized. Patients in the long-acting group had fewer and shorter hospitalizations than other groups during the 1-year post-index period, with a median of one hospitalization for approximately two weeks. Patients receiving rapid-acting insulin or an intensive regimen spent more days in the hospital (a median of 18.8 and 21.0 days, respectively) than those in the long-acting and pre-mixed insulin groups (Table 5).

**Table 5:** Healthcare service utilization (*N =* 2,145)

Healthcare Service (*N =* 2,145)	Long-acting (*N =* 244)	Pre-mixed (*N =* 118)	Rapid-acting (*N =* 997)	Intensive (*N =* 786)
Hospitalization				
Patients hospitalized during the first year, n (%)	80 (32.8)	65 (55.1)	952 (95.5)	691 (87.9)
N of hospitalizations per patient				
Mean (±SD)	2.2 (±1.9)	2.1 (±1.5)	2.7 (±1.9)	2.4 (±1.6)
Median	1.0	2.0	2.0	2.0
Length of stay, days				
Mean (±SD)	20.8 (±27.8)	20.7 (±20.9)	28.0 (±29.2)	27.3 (±23.8)
Median	14.3	16.0	18.8	21.0
Patients re-hospitalized, n (%)	38 (47.5)	37 (56.9)	747 (78.5)	497 (71.9)
Days between two hospitalizations				
Mean (±SD)	103.8 (±101.7)	161.8 (±97.4)	109.3 (±83.9)	104.1 (±82.7)
Median	63.5	159.5	87.0	80.0
Outpatient visits				
Mean (±SD)	20.4 (±25.7)	26.1 (±40.4)	19.9 (±23.5)	22.5 (±26.3)
Median	14.0	13.5	15.0	16.0
Emergency room visits				
Number of visits (all patients)				
Mean (±SD)	0.0 (±0.0)	0.0 (±0.2)	0.0 (±0.2)	0.1 (±0.6)
Median	0.0	0.0	0.0	0.0

The majority of the rapid-acting (78.5 %; *N =* 747) and the intensive regimen groups (71.9 %; *N =* 497) were also rehospitalized during their first year on insulin treatment; fewer patients taking long-acting or pre-mixed therapy were rehospitalized (47.5 % and 56.9 %, respectively). Median days between hospitalizations were longest for those taking pre-mixed insulin (159.5 days); other treatment groups had shorter times with a median of 63.5, 87.0, and 80.0 days for the long-acting, rapid-acting, and intensive regimens, respectively. All groups visited an outpatient clinic approximately once per month (median of 14-16 times per year). Emergency department visits were observed to be rare, with almost no visit to the emergency department across all groups (Table 5).

## Discussion

The primary objective of this study was to describe and evaluate real-world treatment patterns and healthcare service utilization among patients with T2DM in Japan over the 1-year period following initiation of insulin therapy. Few reports describing treatment practice patterns in insulin use among initiators, and via these methods, are available. Although previous studies have investigated the trends in antidiabetic prescription patterns [12, 13], no study has examined different insulin regimens or evaluated healthcare service utilization among those regimen groups. Similarly designed database studies have thus far focused on other patient populations [10] or were conducted in other regions [13]. Nonetheless, automated health resource databases, such as the MDV database, have been found to provide valid and reliable data for pharmacoepidemiologic studies of various diseases [14].

The current study found that the most commonly used insulin therapy among insulin initiators in the database was rapid-acting insulin alone followed by an intensive treatment regimen, which included four different variations of complex regimens. Although the use of long-acting insulin and pre-mixed insulin alone was not frequently observed, most of these patients were taking at least one OAD prior to insulin initiation, thus likely using insulin as a supplement to their antidiabetic therapy. This is consistent with the literature [9]. The higher proportion of rapid-acting insulin compared to long-acting insulin was consistent with the findings of the Cardiovascular Risk Evaluation in people with type 2 Diabetes on Insulin Therapy (CREDIT) study and appeared unique to Japan, as long-acting insulin was observed to be most commonly used as the first insulin in other regions [15]. Moreover, it has been reported that although the overall use of pre-mixed insulin has decreased, the administration of an intensive regimen with rapid-acting and long-acting insulin, as well as bolus (mealtime) therapy using rapid-acting insulin alone, have recently increased [7].

The patient profile of the long-acting and pre-mixed groups appeared to be similar, but the other two groups were different in terms of age and comorbid conditions. Such disparities in patient characteristics among different insulin regimens have been observed previously [12], and a previous study of insulin use patterns among insulin initiators excluded patients using rapid-acting and an intensive regimen to achieve a more homogeneous patient population [13]. Our finding of insulin initiation in an inpatient setting being observed more frequently among those receiving an intensive regimen or rapid-acting insulin may reflect their disease profile being more complicated and requiring specialized care. Previously, initiating insulin as an inpatient was reported among 63.6 % of patients with T2DM regardless of insulin type [16].

The disparities in patient characteristics may also be reflected in our finding of insulin therapy modification. Our finding of insulin modification patterns for patients who received a single type of insulin on their first insulin claim was consistent with the previous studies in other countries which reported modification rates ranging from 5 % [16] over one year to 43.4 % [17] over five years. However, patients who received multiple types of insulin on their first insulin index were more likely to modify their therapy regimen compared with those who received a single type of insulin on the first claim. Because these patients with multiple types of insulin on the first insulin claim belonged to the intensive regimen group (who mostly initiated insulin at an inpatient setting), it is possible that they received closer monitoring and hence were able to switch or add different insulin within a shorter time period.

It is notable that the majority of patients (nearly half of patients receiving an intensive regimen and almost all patients receiving rapid-acting insulin) had a 100-day gap between two insulin claims. This finding was consistent with prior research. An analysis of long-acting and pre-mixed insulin use patterns in the United States found that the majority of insulin initiators (73.5 % taking long-acting insulin and 64.0 % taking pre-mixed insulin) had a 90-day gap between adjacent insulin prescriptions in their first year and were not using insulin “persistently” [13]. Another retrospective database analysis reported that 37.0 % of insulin initiators were not persistent with insulin therapy at 12 months follow-up [18]. However, in the former study, it was reported that almost all (more than 80 %) of those with a 90-day gap restarted insulin after approximately 140 days [13]. Therefore, patients considered “dropped out” from insulin therapy due to our 100-day gap definition in the current study might indeed restart insulin. Nevertheless, more patients appeared to be able to stop insulin therapy when they received rapid-acting insulin or an intensive regimen based on our findings for insulin discontinuation and intermittent use; therefore, although some patient characteristics differed among groups, it is possible that intensive regimens were more effective in relieving glucose toxicity.

Although efforts were made to avoid potential bias associated with only acute-phase treatment by excluding patients who did not continue insulin after discharge from the analyses, it is possible that insulin, especially rapid-acting and regular insulin, which can easily control glucose level, was used for a short period of time in the case of significantly elevated blood glucose during acute-phase treatment of a primary disease or surgery [6, 19]. Such a diverse role of rapid-acting insulin at both specialized and nonspecialized settings has been previously reported [8]. The hospitals included in this analysis were mainly medium or large acute-phase hospitals with specialized physicians, and patients receiving rapid-acting insulin or an intensive regimen were observed to have more complications, likely requiring specialized care. In addition, given the length of hospital stay observed in the current study, it is possible that patients received rapid-acting insulin alone or as a part of an intensive regimen for educational/training purposes. Local guidelines recommend some patients initiate insulin therapy in-hospital and stay for approximately 7 to 14 days to acclimate to the new insulin regimen [6], and an intensive regimen has been proven effective among those initiating insulin through this educational hospitalization program [20].

Although enabling the powerful and relatively efficient evaluation of large swaths of healthcare data, the methods of the current study were not without their limitations. First, the database examined did not allow for the linkage of diagnosis and prescription data directly; therefore, it was difficult to ensure patients’ utilization of insulin was truly for diabetes rather than glucose toxicity [19]. Second, due to the nature of administrative hospital-based databases, the database only reflected medium- and large-sized administrative hospitals and did not include outpatient clinics. It was also not possible to follow those patients who went to a different hospital or clinic or to identify the same patient receiving care at more than one hospital. This reduced the generalizability of the data because the data collected were restricted to reflect clinical practice at larger hospital settings and patients who returned to the same hospital or clinic for continuous care. Third, the sample size was relatively small after only including patients with three insulin claims and excluding those patients who did not continue taking insulin after hospitalization. However, these restrictions were important, providing more confidence that the population analyzed was truly diabetic and not simply receiving insulin for other reasons. Fourth, treatment patterns of insulin initiators were complicated; therefore, it is possible that classifying insulin regimen group based on claims over the year resulted in misclassification for some cases. As seen in our modification analysis, three of the groups added or changed treatment within a median of 30 days. Therefore, it is possible that they were misclassified into a more intensive treatment regimen when they only switched to a different class of therapy. Additionally, insulin daily dose is not reliable because total volume is provided but the duration is not; therefore, the time between prescriptions was used as a proxy for monthly or 3-month prescriptions. Finally, the database did not provide key clinical information that would determine clinical decision making on types of insulin regimen such as body mass index (BMI), duration of disease, hypoglycemia inpatient admission, or useful days supply for insulin prescriptions. The patient profile across groups may have differed due to these unobserved factors or information unavailable in the database. For example, previous studies have reported that age at diagnosis, disease duration, and BMI were associated with clinical outcomes [7, 21] and that disease duration and daily insulin dosage at the initiation were associated with insulin discontinuation [22]. We recommend careful interpretation of the current findings.

## Conclusions

This is the first multi-center retrospective database study to delineate the real-world insulin utilization patterns and healthcare service use among all types of insulin initiators with T2DM in Japan. Variation in patient characteristics and insulin utilization patterns across insulin groups were revealed, particularly between the long-acting/pre-mixed groups and the rapid-acting/intensive regimen groups, in terms of age and comorbid conditions. Healthcare services were found to both diverge (e.g., hospitalization) and remain similar (e.g., emergency department visits) among the groups. Although additional research to further delineate the factors influencing insulin use and regimen initiation remains warranted, the current study provides valuable insight into the real-world insulin and healthcare service utilization patterns in Japan. Future research should assess the basis for these various utilization patterns associated with insulin to conduct robust analyses of clinical and economic outcomes. The detailed treatment patterns and healthcare service use by insulin regimen provides a foundation for future research to build upon.

## Additional file

Additional file 1: Table S1. Classification of insulin. (DOCX 79 kb)

## Abbreviations

ICD-10: International Classification of Disease 10th revision
JDS: Japan Diabetes Society
MDV: Medical Data Vision Co., Ltd
NGSP: National Glycohemoglobin Standardization Program
T2DM: Type 2 Diabetes Mellitus

## Footnotes

1 Excludes those living in the areas severely affected by the 2011 Great East Japan Earthquake.

2 7.4 % HbA1c was calculated based on 7.0 % JDS

3 9.4 % HbA1c was calculated based on 9.0 % JDS
