# Supplementary material for: Utilization patterns of insulin therapy and healthcare services among Japanese insulin initiators during their first year: a descriptive analysis of administrative hospital data
Source: BMC Health Serv Res. 2016 Jan 12;16:6. doi: 10.1186/s12913-016-1264-2 (PMC4710987; doi:10.1186/s12913-016-1264-2)
Supplement: Additional file 1: Table S1. — Classification of insulin. (DOCX 79 kb) [file 12913_2016_1264_MOESM1_ESM.docx]

**Additional file 1: Table S1. Classification of Insulin**

| **Insulin Group** | **Analogue or human** | **Generic name** | **Product Name and Dose** | **Drug Pricing Listing Code** | **Receipt Code** |
| --- | --- | --- | --- | --- | --- |
| Rapid-acting insulin | Analogue | Insulin Glulisine | Apidra, 100 unit/mL | 2492418A1024 | 621911101 |
|  |  |  | Apidra Cart, 300 unit | 2492418A2020 | 621911301 |
|  |  |  | Apidra SoloSTAR, 300 unit | 2492418G1027 | 621911201 |
|  |  | Insulin Aspart | NovoRapid, 100 unit/mL | 2492415A3031 | 620008895 |
|  |  |  | NovoRapid InnoLet, 300 unit | 2492415G3026 | 621926901 |
|  |  |  | NovoRapidFlexPen, 300 unit | 2492415G1031 | 620008893 |
|  |  |  | NovoRapid Penfill, 300 unit | 2492415A2035 | 620008894 |
|  |  |  | NovoRapid Vial, 100 unit/mL | 2492415A3023 | 640451038 |
|  |  |  | NovoRapid, 300 unit | 2492415A2027 | 640451040 |
|  |  |  | NovoRapid FlexPen, 300 unit | 2492415G1023 | 640451041 |
|  |  | Insulin Lispro | Humalog, 100 unit/mL | 2492414A2030 | 620008916 |
|  |  |  | Humalog Cart, 300 unit | 2492414A1026 | 640451027 |
|  |  |  | Humalog Kit, 300 unit | 2492414G1029 | 640451028 |
|  |  |  | Humalog Vial, 100 unit/mL | 2492414A2022 | 640451029 |
|  |  |  | Humalog Miriopen, 300 unit | 2492414G5024 | 620007460 |
|  | Human | Insulin Human (biosynthesis) | InnoLet R, 300 unit | 2492413G8045 | 640453021 |
|  |  |  | Novolin R, 300 unit | 2492403A4043 | 620008897 |
|  |  |  | Novolin R, 100 unit/mL | 2492403A4035 | 642490107 |
|  |  |  | Novolin R FlexPen | 2492413G8053 | 620000265 |
|  |  | Insulin Human (genetical recombination) | Humacart  R, 300 unit | 2492403H4025 | 640407220 |
|  |  |  | Humacart  R, 300 unit | 2492413G8037 | 640422068 |
|  |  |  | Humulin R, 100 unit/mL | 2492403A4051 | 620008909 |
|  |  |  | Humulin R U-100, 100 unit | 2492403A4027 | 642490059 |
|  |  |  | Humulin R Cart, 300 unit | 2492403H4041 | 620008907 |
|  |  |  | Humulin R Kit, 300 unit | 2492413G8061 | 620008908 |
|  |  |  | Humulin R Miriopen, 300 unit | 2492413P2020 | 622114401 |
|  |  |  | Penfill R, 300 unit | 2492403H4050 | 620008932 |
|  |  |  | Penfill R, 300 unit | 2492403H4033 | 640422074 |
| Pre-mixed insulin | Analogue | Pre-mix Insulin Aspart | NovoRapid 30 Mix, 300 unit | 2492415A4020 | 620000447 |
|  |  |  | NovoRapid 30 Mix Penfill, 300 unit | 2492415A4038 | 620008896 |
|  |  |  | NovoRapid 30 Mix FlexPen, 300 unit | 2492415G2020 | 620000448 |
|  |  |  | NovoRapid 50 Mix FlexPen, 300 unit | 2492415G4022 | 621973201 |
|  |  |  | NovoRapid 70 Mix FlexPen, 300 unit | 2492415G5029 | 621973301 |
|  |  | Pre-mixed Insulin Lispro | Humalog Mix 25 Cart, 300 unit | 2492414A3029 | 620002439 |
|  |  |  | Humalog Mix 25 Kit, 300 unit | 2492414G2025 | 620002442 |
|  |  |  | Humalog Mix 25 Miriopen, 300 unit | 2492414G6020 | 620007461 |
|  |  |  | Humalog Mix 50 Cart, 300 unit | 2492414A4025 | 620002440 |
|  |  |  | Humalog Mix 50 Kit, 300 unit | 2492414G3021 | 620002443 |
|  |  |  | Humalog Mix 50Miriopen, 300 unit | 2492414G7027 | 620007462 |
|  | Human | N.A. | InnoLet 30R, 300 unit | 2492413G5046 | 640453023 |
|  |  |  | InnoLet 40R, 300 unit | 2492413G6034 | 620000204 |
|  |  |  | InnoLet 50R, 300 unit | 2492413G7030 | 620000205 |
|  |  |  | Novolin 10R FlexPen, 300 unit | 2492413G3043 | 620000267 |
|  |  |  | Novolin 20R FlexPen, 300 unit | 2492413G4040 | 620000268 |
|  |  |  | Novolin 30R, 100 unit/mL | 2492413A4039 | 620008899 |
|  |  |  | Novolin 30R 100, 100 unit | 2492413A4020 | 642490121 |
|  |  |  | Novolin 30R FlexPen, 300 unit | 2492413G5054 | 620000269 |
|  |  |  | Novolin 40R FlexPen, 300 unit | 2492413G6042 | 620000270 |
|  |  |  | Novolin 50R FlexPen, 300 unit | 2492413G7049 | 620000271 |
|  |  |  | Humacart 3/7, 300 unit | 2492403H6028 | 640407222 |
|  |  |  | Humacart 3/7, 300 unit | 2492413G5038 | 640422067 |
|  |  |  | Humulin 3/7, 100 unit/mL | 2492403H1034 | 620008915 |
|  |  |  | Humulin 3/7 U-100 | 2492403H1026 | 640406239 |
|  |  |  | Humulin 3/7 Cart, 300 unit | 2492403H6036 | 620008913 |
|  |  |  | Humulin 3/7 Kit, 300 unit | 2492413G5062 | 620008914 |
|  |  |  | Humulin 3/7 Miriopen, 300 unit | 2492413G9025 | 622114601 |
|  |  |  | Penfill 20R, 300unit | 2492413H6021 | 640412081 |
|  |  |  | Penfill 30R, 300unit | 2492413H7036 | 620008934 |
|  |  |  | Penfill 30R, 300unit | 2492413H7028 | 640412082 |
|  |  |  | Penfill 40R, 300unit | 2492413H8032 | 620008935 |
|  |  |  | Penfill 40R, 300unit | 2492413H8024 | 640412083 |
|  |  |  | Penfill 50R, 300unit | 2492413H9039 | 620008936 |
|  |  |  | Penfill 50R, 300unit | 2492413H9020 | 640412084 |
| Long-acting insulin | Analogue | Neutral Protamine Insulin Lispro | Humalog N Cart, 300 unit | 2492414A5021 | 620002441 |
|  |  |  | Humalog N Cart, 300 unit | 2492414G4028 | 620002444 |
|  |  |  | Humalog N Cart, 300 unit | 2492414G8023 | 620007459 |
|  |  | Insulin Glargine | Lantus, 100 unit/mL | 2492416A3036 | 620008945 |
|  |  |  | Lantus Opti-click, 300 unit | 2492416A2030 | 620008942 |
|  |  |  | Lantus Opti-click, 300 unit | 2492416A2021 | 620002445 |
|  |  |  | Lantus Cart, 300 unit | 2492416A1033 | 620008943 |
|  |  |  | Lantus Cart, 300 unit | 2492416A1025 | 620000442 |
|  |  |  | Lantus Kit, 300 unit | 2492416G1036 | 620008944 |
|  |  |  | Lantus Kit, 300 unit | 2492416G1028 | 620000443 |
|  |  |  | Lantus SoloSTAR, 300 unit | 2492416G2024 | 620007536 |
|  |  |  | Lantus Vial, 100 unit | 2492416A3028 | 620004781 |
|  |  | Insulin Detemir | Levemir InnoLet, 300 unit | 2492417G2029 | 621927001 |
|  |  |  | Levemir FlexPen, 300 unit | 2492417G1030 | 620008952 |
|  |  |  | Levemir Penfill, 300 unit | 2492417A1038 | 620008953 |
|  |  |  | Levemir, 300 unit | 2492417A1020 | 620005900 |
|  |  |  | Levemir FlexPen, 300 unit | 2492417G1022 | 620005901 |
|  | Human | Neutral Protamine Hagedorn (NPH) | InnoLet N, 300 unit | 2492413G1040 | 640453022 |
|  |  |  | Novolin N, 100 unit/mL | 2492413A2036 | 620008898 |
|  |  |  | Novolin N 100, 11 unit | 2492413A2028 | 642490123 |
|  |  |  | Novolin N FlexPen, 300 unit | 2492413G1059 | 620000266 |
|  |  |  | Humacart N, 300 unit | 2492403H5021 | 640407221 |
|  |  |  | Humacart N, 300 unit | 2492413G1032 | 640422069 |
|  |  |  | Humulin N, 100 unit/mL | 2492403A3047 | 620008912 |
|  |  |  | Humulin N U-100, 100 unit | 2492403A3020 | 642490061 |
|  |  |  | Humulin N Cart, 300 unit | 2492403H5030 | 620008910 |
|  |  |  | Humulin N Kit, 300 unit | 2492413G1067 | 620008911 |
|  |  |  | Humulin N Miriopen, 300 unit | 2492413P1024 | 622114501 |
|  |  |  | Penfill N, 300 unit | 2492413H4037 | 620008933 |
|  |  |  | Penfill N, 300 unit | 2492413H4029 | 640412085 |
